# Carnitine palmitoyltransferase 1 (CPT1) alleviates oxidative stress and apoptosis of hippocampal neuron in response to beta-Amyloid peptide fragment Aβ_25-35_

**DOI:** 10.1080/21655979.2021.1967032

**Published:** 2021-08-23

**Authors:** Yiyun Ding, Hongxia Zhang, Zhaojun Liu, Qiuping Li, Yujiao Guo, Ye Chen, Yue Chang, Hongyan Cui

**Affiliations:** a Department of Geriatrics, The Second Hospital of Tianjin Medical University, Tianjin, China; b Laboratory Division, Ankang Center for Disease Control and Prevention, Ankang, Shaanxi Province, China

**Keywords:** Aβ, CPT1C, Alzheimer’s disease, hippocampal neurons

## Abstract

CPT1C, which is expressed in hippocampus, influences ceramide level, endogenous cannabinoid and oxidation process, as well as plays an important role in various brain functions such as learning. This study aimed to investigate the role of CPT1C in Alzheimer’s disease (AD) and its underlying mechanism. We established a model of Alzheimer’s disease in vitro by exposing primary hippocampal neurons to beta-Amyloid peptide fragment 25–35 (Aβ_25-35_). The cell viability, lactate dehydrogenase (LDH) level, expressions of reactive oxygen species (ROS), malondialdehyde (MDA) and superoxide dismutase (SOD) were detected using Cell Counting Kit-8 (CCK-8), LDH assay, ROS kits, malondialdehyde (MDA) kits and SOD kits, respectively. Moreover, the expression of oxidative stress-related proteins as well as the expressions of amyloid precursor protein (App), p-Tau andβ-site APP-cleaving enzyme1 (Bace-1) were measured using quantitative reverse transcription PCR (RT-qPCR) and western blot. Tunel and western blot were adopted to detect apoptosis as well as its related proteins. After the treatment of peroxisome proliferators-activated receptor alpha (PPARα), CPT1C expression was detected with the application of RT-qPCR and western blot. CPT1C expression was reduced in Aβ_25-35_-induced HT22 cells. Overexpression of CPT1C relieved cell viability and toxic injury as well as attenuated oxidative stress, apoptosis and expression levels of AD marker proteins. Moreover, higher doses of PPARα agonist activate the expression of CPT1C in Aβ_25-35_-induced HT22 cells. In conclusion, CPT1C alleviates Aβ_25-35_-induced oxidative stress, apoptosis and deposition of AD marker proteins in hippocampal neurons, suggesting that CPT1C has favorable effects on alleviating AD and participates in PPARα activation.

## Introduction

Alzheimer’s disease (AD) is referred to as progressive cognitive decline resulting in dementia [1]. According to recent statistics, around 35.6 million people have suffered from dementia in the globe, which may be partially due to the high prevalence of AD [2]. What is beyond people’s recognition is that neurodegenerative disease is not confined to the elder but is spreading quickly,, resulting in increased financial burden and lowering people’s life quality [3]. Current therapeutic methods, including treatment with cholinesterase inhibitors, cannot change the rising trend of illnesses [4].

Carnitine palmitoyl transferase (CPT) system, a multiprotein complex featuring catalytic properties is located in a core represented by CPT1 and CPT2 in the outer and inner membrane of the mitochondria, respectively [5]. Similar to the canonical CPT enzymes in the sequence of primary amino acid, CPT1C is located in the endoplasmic reticulum of neurons and exerts residual catalytic effects in vitro with palmitoyl-CoA [6,7]. As evidenced by previous studies, depletion of CPT1C made more palmitoyl-CoA available, and mice with CPT1C knockdown exhibited metabolic disturbance like impaired gluconeogenesis and muscle glucose uptake [8]. CPT1C, which is expressed in hippocampus, influences ceramide level, endogenous cannabinoid and oxidation process, and plays an important role in various brain functions such as learning [5]. In addition, some studies held that the deficiency of CPT1C on brain can cause motor dysfunction and behavioral defects [9]. CPT1C is considered to possibly involve in AD due to its role in energy homeostasis. The onset of AD is associated with insulin resistance, which is modulated by palmitate in the hypothalamus and implicated in increased production of reactive oxygen species (ROS) [5,10–12]. However, the role of CPT1C in AD has remained elusive, which constitutes the pivotal focus of our study.

In the present study, we hypothesize that CPT1C plays a role in AD and is involved in the regulation for oxidative stress, apoptosis and AD markers. The study was designed to decipher the role of CPT1C in an in vitro model of AD and figure out how CPT1C was implicated in AD.

## Materials and methods

### Cell culture and treatment

Mouse hippocampal neuron (HT22) was provided from Salk Institute (La Jolla, CA, USA). The cells were cultured in Dubelcco’s modified eagle medium (DMEM; Gibco, NY, USA) containing 10% fetal bovine serum (FBS; Gibco, NY, USA) and 1% penicillin/streptomycin at 37 C in a humidified incubator with 5% CO_2_. Aβ_25-35_ was diluted to 1 mM with sterilized saline water and then incubated at 37 C for 7 days before use, as previously described [13]. Thereafter, the HT22 cells were treated with 20 mol/L Aβ_25-35_ for 48 h. CPT1C overexpression plasmids (Ov-CPT1C, 1 µg/mL) and its empty vector (Ov-NC) were obtained from Shanghai Integrated Biotech Solutions Co., Ltd (Shanghai, China), and transfection was conducted for 24 h by Lipofectamine 3000 (Invitrogen). Gemfibrozil (Abcam, ab142883), a PPARa agonist [14], was used for the following experiments.

### RT-qPCR

RNA from HT22 cells was extracted using a TRIzol® kit in accordance with the manufacturer’s instructions. The synthesization with complementary DNA (cDNA) was performed using a PrimeScript RT Master Mix kit (Takara Biotechnology, Co., Ltd.). Gene expression was quantified by quantitative real-time PCR (RT-qPCR) on ABI 7500 PCR system (ABI, USA) using SYBR Green PCR Master Mix. All samples were measured in triplicate and the mean value was calculated. Quantitative measurements were determined using 2^−ΔΔCT^ method [15], and expression of GAPDH was considered as the internal control.

### Western blot

Total proteins were isolated from HT22 cells using RIPA buffer (Beyotime, Shanghai, China). A BCA kit was used to quantify the concentration of protein samples. Subsequently, equal amounts of proteins were subjected to 10% SDS-PAGE gel, followed by the transfer to PVDF membranes (EMD Millipore, Billerica, MA, USA). After being blocked with 5% nonfat milk, the membranes were then incubated with primary antibodies (CPT1C, cat.no. DF12150; SOD1, cat.no. AF5198; SOD2, cat.no. AF5144; Bcl2, cat.no. AF6139; Bax, cat.no. AF0120; cleaved PARP, cat.no. BF9106; APAF-1, cat.no. AF0117; App, cat.no. AF6084; p-Tau, cat.no. AF3148. Affinity, USA) (Bace-1, cat.no. ab183612; GAPDH cat.no. ab8245. Abcam). Thereafter, these blots were incubated with a HRP-conjugated antibody (cat.no. #7074, Cell Signaling Technology, Inc.) at 37°C for 1 h. Proteins were visualized with an enhanced chemiluminescence (ECL) and the expression of protein was normalized to GAPDH.

### CCK-8 assay

Briefly, cells were inoculated into 96-well plates at a concentration of 3 × 10^4^/well for 12 h. After indicated treatment, CCK-8 reagent (abcam, England) was added to each well to incubate the cells for another 4 h. Then, the optical density (OD) value at 450 nm was determined using a microplate reader (Molecular Devices, San Jose, CA, United States).

Detection of Superoxide Dismutase (SOD) Activity, Malondialdehyde (MDA) Content, and LDH Release Assay

For the detection of cell SOD activity and MDA content, cells after treatment were cultured in 96-well plates, and then sonicated and centrifuged to obtain the supernatant. The SOD activity and MDA content were then measured according to the manufacturer’s protocol (Beyotime, Shanghai, China) and results were displayed as a multiple relative to the control group. The cell supernatant was collected and used for the determination of LDH activities using LDH Cytotoxicity Assay Kit according to manufacturer’s protocol (Beyotime, Shanghai, China).

### Measurement of ROS

The fluorescent probe 2′,7′-dichlorfluorescein-diacetate (DCFH-DA) kit was used for the detection of ROS level in HT22 cells according to manufacturer’s guidance (Beyotime, Shanghai, China). Briefly, the cells were seeded in 96-well plates and then processed with working solution for 20 min at 37°C. After washing with serum-free medium for three times, the fluorescence density was detected using a fluorescence microplate (biosys, Germany).

### TUNEL assay

The cell apoptosis rate was determined using TUNEL assay kits (Invitrogen; Thermo Fisher Scientific). Briefly, the collected cells were fixed with 4% paraformaldehyde for 30 min incubated with 0.3% Triton X-100 in PBS for 10 min according to the manufacturer’s protocol. After staining with DAPI for 30 min, the positive-apoptotic cells were counted under a magnification of 200 × .

### Statistical analysis

Statistical analysis was performed using Graph Pad Prism 6 (Graph Pad Software, La Jolla, CA). The results were expressed as mean ± standard deviation (SD). One-way ANOVAwas used for comparison among multiple groups, followed by Tukey’s post hoc test between two groups. P < 0.05 indicated statistical significance. Each experiment was repeated at least three times.

## Results

CPT1C expression was reduced in Aβ_25-35_-induced HT22 cells

**Figure 1.**
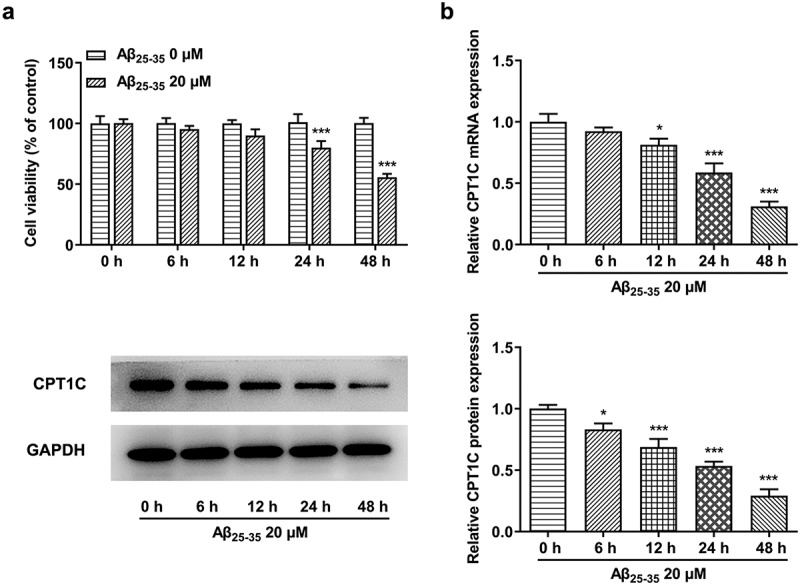
CPT1C expression was reduced in Aβ_25-35_-induced HT22 cells. HT22 cells were treated with Aβ_25-35_ (20 μM) for 6 h, 12 h, 24 h or 48 h, respectively

Despite the fact that CPT1C is ubiquitously presented in various transformed cells and cancers [16], the current strategy we used for the following study is to detect its expression in vitro. Firstly, we induced the cells by 20 µM Aβ_25-35_, while the control group was not treated with Aβ_25-35_. Results from CCK-8 clearly implied that the cell viability of Aβ_25-35_-induced HT22 cells was decreased and the effects of A_25-35_ on HT22 cells were in a time-dependent manner (Figure 1(a)). As Figure 1(b) shown, the expression of CPT1C was decreased both at a trascript and at a protein level by Aβ_25-35_ over time.

(a) The cell viability of Aβ_25-35_-induced HT22 cells was detected using CCK-8 assay. (b) mRNA and protein expressions of CPT1C in Aβ_25-35_-induced HT22 cells were measured using RT-qPCR and western blot, respectively. *P < 0.05 and ***P < 0.001 vs. Control group.

CPT1C overexpression attenuated cell viability and toxic injury in Aβ_25-35_-induced HT22 cells

**Figure 2.**
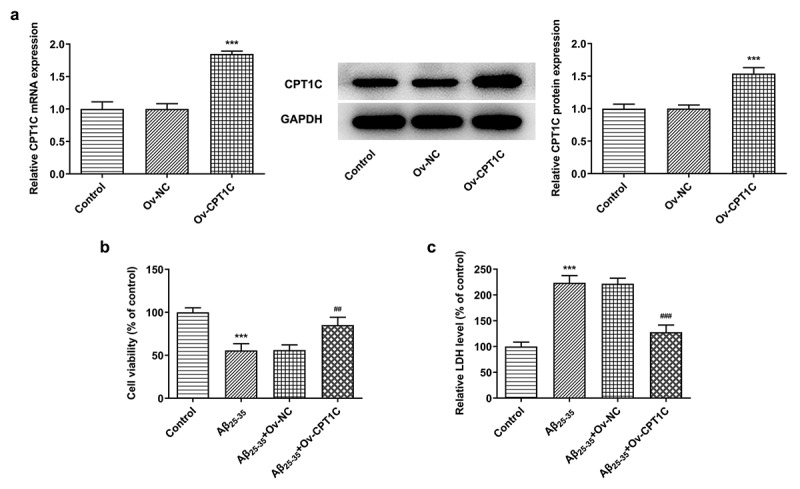
CPT1C overexpression attenuated cell viability and toxic injury in Aβ_25-35_-induced HT22 cells. HT22 cells were transfected with Ov-CPT1C or Ov-NC for 24 h, and then treated with Aβ_25–35_ for another 24 h

To determine the role of CPT1C, we transfected Aβ_25-35_-induced HT22 cells with CPT1C overexpression plasmids to observe whether CPT1C expression could manipulate the cellular behaviors in hippocampal neurons. As Figure 2(a) indicated, the expression of CPT1C was greatly increased after Aβ_25-35_ induction. Results from CCK-8 assay indicated that the decreased cell viability of HT22 cells induced by Aβ_25-35_ was reverted by CPT1C overexpression (Figure 2(b)). Furthermore, the relative LDH level in HT22 cells was significantly increased after Aβ_25-35_ induction whileCPT1C overexpression reversed the promotive effects of Aβ_25-35_ (Figure 2(c)). The above results illustrated that the overexpression of CPT1C attenuated cell viability and toxic injury in Aβ_25-35_-induced HT22 cells.

(a) mRNA and protein expressions of CPT1C were measured using RT-qPCR and western blot, respectively. ***P < 0.001 vs Ov-NC. (b) The cell viability of Aβ_25-35_-induced HT22 cells was detected using CCK-8. ***P < 0.001 vs. Control group, ^##^P < 0.01 vs Aβ_25-35_ + Ov-NC. (c) The relative LDH level of Aβ_25-35_-induced HT22 cells was checked using LDH assay. ***P < 0.001 vs. Control group, ^###^P < 0.001 vs Aβ_25-35_ + Ov-NC.

CPT1C overexpression attenuated oxidative stress in Aβ_25-35_-induced HT22 cells

**Figure 3.**
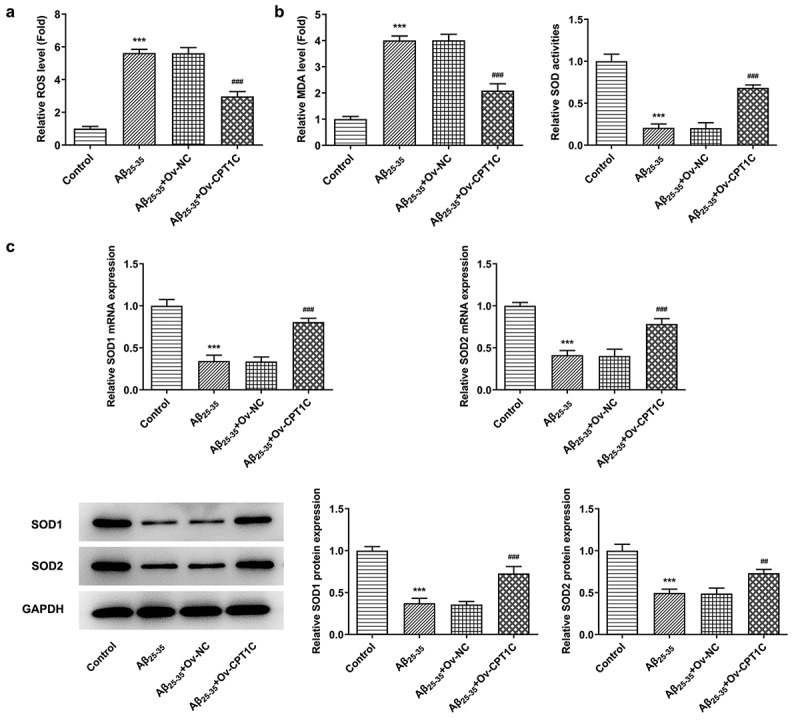
CPT1C overexpression attenuated oxidative stress in Aβ_25-35_-induced HT22 cells. Following transfection of Ov-CPT1C or Ov-NC for 24 h, HT22 cells were treated with Aβ_25–35_ for another 24 h

We then observed if oxidative stress and cell apoptosis were changed after CPT1C was overexpressed in A_25-35_-induced HT22 cells. According to Figure 3(a), the greatly increased levels of ROS and MDA, as well as in Aβ_25-35_-induced HT22 cells was suppressed by CPT1C overexpression. In addition, CPT1C overexpression downregulated MDA levels in Aβ_25-35_-induced HT22 cells but enhanced SOD activities. Besides, SOD1 expression levels at transcription and translation levels were increased by CPT1C overexpression when compared with the cotreatment group of Aβ_25-35_ and Ov-NC. These suggested that CPT1C overexpression exhibited inhibitory effects on oxidative stress in Aβ_25-35_-induced HT22 cells (Figure 3(b,c)).

(a) ROS expression was detected using ROS kits. (b) MDA levels and SOD activity were measured using MDA and SOD kits, respectively. (c) The relative mRNA and protein expression of SOD1 and SOD2 in A_25-35_-induced HT22 cells were measured using RT-qPCR and western blot, respectively. ***P < 0.001 vs. Control group, ^##^P < 0.01 and ^###^P < 0.001 vs A_25-35_ + Ov-NC.

CPT1C overexpression decreased the apoptosis of Aβ_25-35_-induced HT22 cells

**Figure 4.**
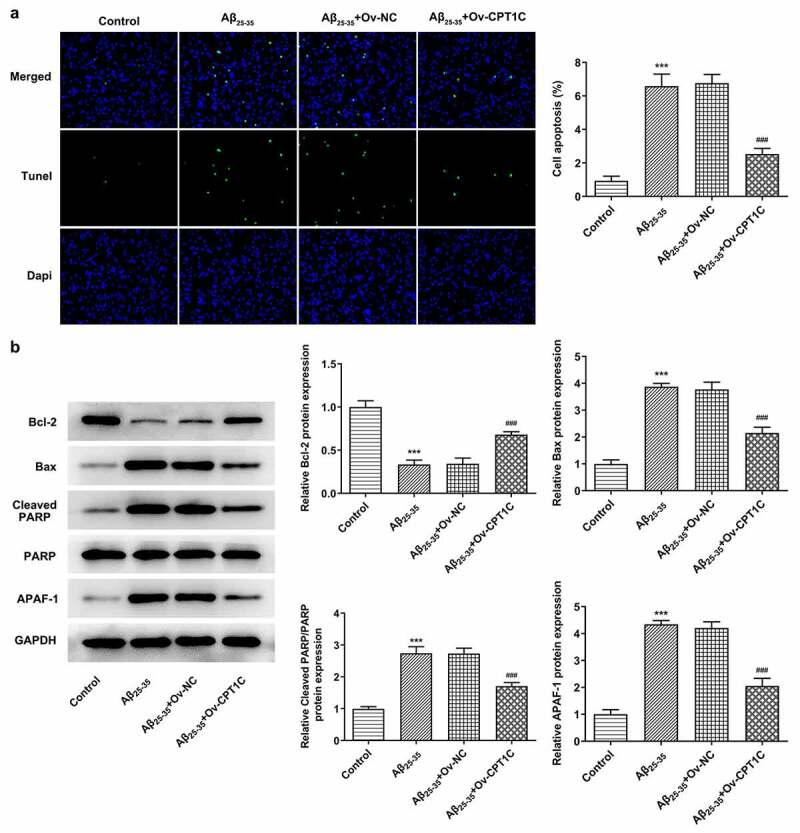
CPT1C overexpression decreased the apoptosis of Aβ_25-35_-induced HT22 cells. Following transfection of Ov-CPT1C or Ov-NC for 24 h, HT22 cells were treated with Aβ_25–35_ for another 24 h

We further determined whether CPT1C regulated apoptosis in HT22 cells treated with Aβ_25-35._ As Figure 4(a) demonstrated, the apoptosis in HT22 cells was hugely increased by Aβ_25-35_ induction in comparison with the Aβ_25-35_ group. However, the increased apoptosis level was then decreased by CPT1C overexpression. Moreover, Aβ_25-35_ brought about Bcl2 downregulation and Bax, cleaved PARP, and APAF-1 upregulation, which were then reversed by CPT1C overexpression (Figure 4(b)). Therefore, we could conclude that overexpression of CPT1C attenuates the apoptosis in hippocampal neurons.

(a) The apoptosis of Aβ_25-35_-induced HT22 cells was evaluated by TUNEL. (b) The expressions of Bcl2, Bax, cleaved PARP and APAF-1 were measured by western blot. ***P < 0.001 vs. Control group, ^###^P < 0.001 vs A_25-35_ + Ov-NC.

CPT1C overexpression decreased the deposition of AD marker proteins in Aβ_25-35_-induced HT22 cells

**Figure 5.**
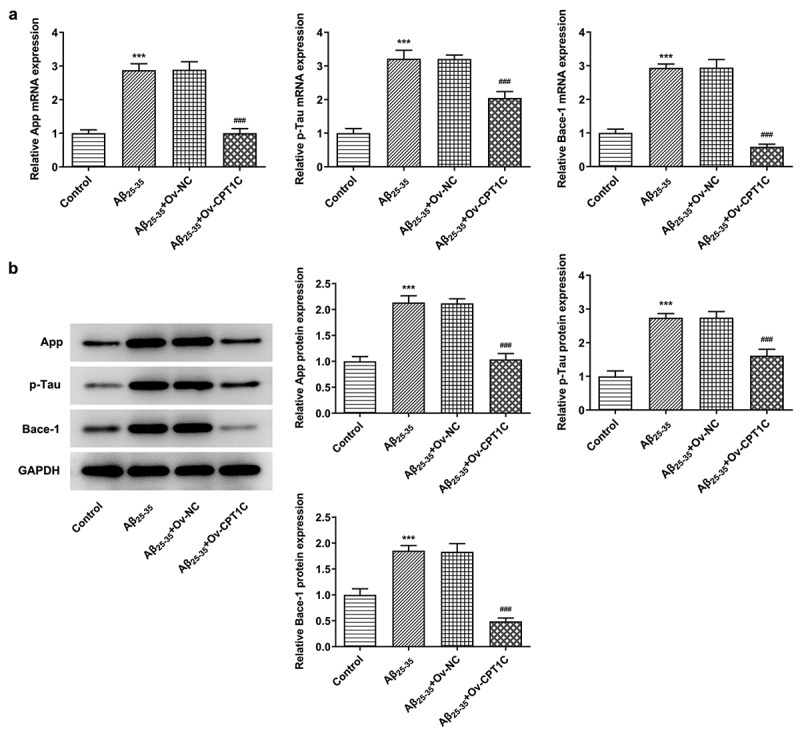
CPT1C overexpression decreased the deposition of AD marker proteins in Aβ_25-35_-induced HT22 cells. Following transfection of Ov-CPT1C or Ov-NC for 24 h, HT22 cells were treated with Aβ_25–35_ for another 24 h

We next analyzed whether CPT1C overexpression could change the expression of AD markers in Aβ_25-35_-induced HT22 cells. With the application of RT-qPCR and western blot, the relative mRNA and protein expressions of App, p-Tau and Bace-1 were measured. Compared with Control, the expressions of App, p-Tau and Bace-1 were greatly upregulated by Aβ_25-35_, while CPT1C overexpression reversed the promotive effects of Aβ_25-35_ on deposition of AD marker proteins, evidenced by the downregulated expressions of App, p-Tau and Bace-1 in contrast with Aβ25-35 + Ov-NC (Figure 5(a,b)). PPARα activation could increase CPT1C expression in Aβ_25-35_-induced HT22 cells

(a) The mRNA expressions of App, p-Tau and Bace-1 were evaluated using RT-qPCR. (b) The protein expressions of App, p-Tau and Bace-1 were evaluated using western blot. ***P < 0.001 vs. Control group, ^###^P < 0.001 vs Aβ_25-35_ + Ov-NC.

**Figure 6.**
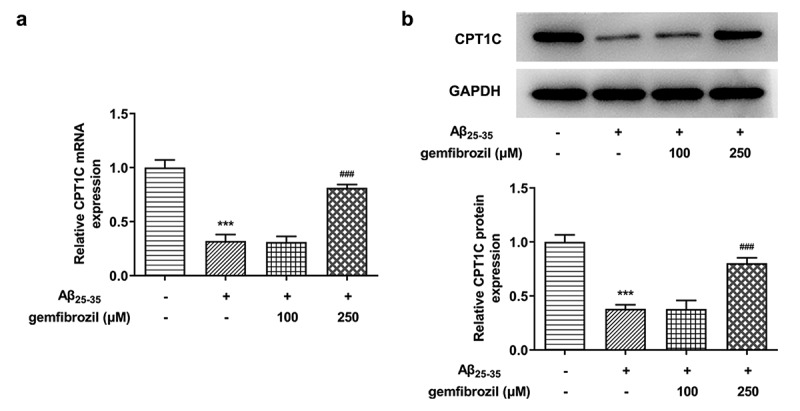
PPARα activation could increase CPT1C expression in Aβ_25-35_-induced HT22 cells. HT22 cells were co-treated with gemfibrozil 100 μM or 250 μM, and Aβ_25-35_ for 48 h

In order to explore whether CPT1C could be activated by PPARα, gemfibrozil, an agonist of PPARα, was used to treat Aβ_25-35_-induced HT22 cells. According to Figure 6(a,b), the expression of CPT1C was significantly diminished by Aβ_25-35_ in comparison with Control. Studies have noted that PPARα regulates the malignant behaviors of tumor cells by targeting CPT1C, and PPARα activation can alleviate the amyloidosis and reverse memory deficits and anxiety in AD [14,17]. After the treatment of gemfibrozil with a dose of 250 μM, mRNA and protein expressions of CPT1C gained a huge growth, revealing that PPARα activation could increase CPT1C expression in Aβ_25-35_-induced HT22 cells.

(a) The relative CPT1C mRNA expression in Aβ_25-35_-induced HT22 cells was detected using RT-qPCR. (b) The protein expression of CPT1C expression in Aβ25-35-induced HT22 cells was detected using western blot. ***P < 0.001 vs. Control group, ^###^P < 0.001 vs Aβ_25-35_+ gemfibrozil 100 μM.

## Discussion

AD is a neurodegenerative disorder that affects the cognitive functions of human beings, especially the elderly [18]. The aggregation of Aβ, which has been considered as a major driver of AD progression, was linked to the severity of cognitive deficits [19,20]. Similar to the induction procedures conducted by previous studies, this paper used Aβ_25-35_ to construct the *in vitro* AD model.

The intricate pathology of AD has necessitated novel targeted treatment methods to fight against its invasion [21]. Oxidative stress is a major driver of AD pathophysiology, and antioxidant agents have been highlighted as optimal choices for the inhibition of AD progression [22]. Patients with AD are often found to suffer from oxidative damage to the neuronal tissues [23]. In this study, CPT1C overexpression led to increased SOD expression, and decreased MDA as well as ROS expression in Aβ25-35-induced HT22 cells. A previous study showed that the loss of CPT1C triggered increased sensitivity of cells to oxidative stress, as demonstrated by the elevation of ceramides, a major regulator of oxidative stress [24]. The findings in this study were consistent with the above-mentioned study. Mounting evidence has noted that apoptosis, which is also called programmed cell death, partakes in the AD-associated nerve cell death [25,26]. Thus, the changes of apoptosis were observed after CPT1C was overexpressed in Aβ_25-35_-induced HT22 cells. Importantly, the apoptosis elevated by Aβ_25-35_ was attenuated by CPT1C overexpression. Moreover, it was found that overexpression of CPT1C abolished the promotive effects of Aβ_25-35_ on enhancing the expression of AD markers. Thus, decreased CPT1C by Aβ_25-35_ treatment was able to induce oxidative stress and apoptosis, and CPT1C could be involved in AD pathology.

Peroxisome proliferators activated receptors (PPARs) are a family of ligand-regulated nuclear receptors regulating transcriptions via a complex mechanism [27–29]. Evidence has shown that apart from the modulation of mitochondria metabolism, PPARα participates in amyloid beta precursor protein (APP) in the brain and it may also influence Tau protein phosphorylation via A [30]. It is well acknowledged that PPARαplayed an essential role in neuronal cells [31]. Moreover, PPARα polymorphism may be deemed as a risk factor for AD [32]. Thus, we speculated that there might be a close connection between CPT1C and PPARα in regulating the progression of AD. The administration of gemfibrozil at higher dosage increased the expression of CPT1C inhibited by Aβ_25-35_, which tallied with the idea proposed by several experts that alteration of PPARα signaling may lead to activation of APP metabolism that contributed to AD pathogenesis [30]. Meanwhile, some held that the activation of PPARα, which was found to be downregulated in AD brain, may also alleviate the inducive effects of Aβ_25-35_ on AD [30]. Besides, the recognized association of PPARα and CPT1C in our study was also found by other study which revealed that PPARα was able to activate the transcription of CPT1C promoter to regulate cell proliferation [17]. Taken together, the inhibition of PPARα in HT22 cells under Aβ_25-35_ stimulation is the most cause of decreased CPT1C expression, further inducing oxidative stress and apoptosis.

## Conclusion

Aβ25-35 induced decreased CPT1C, thereby leading to oxidative stress and apoptosis. The decreased CPT1C could be due to the inhibition of Aβ25-35 for PPARα. CPT1C could play a vital role in AD and may provide insight into AD treatment.

## Limitation

The limitation is that current therapeutic strategies can only mildly slow but not halt the progression of AD and this paper has suggested favorable outcomes of CPT1C in alleviating AD and its underlying mechanism related to PPAR activation. It is noteworthy that further in vitro and vivo, and clinical studies regarding the role of CPT1C in AD are still needed for the better management of this disorder.

## Highlights

1. HT22 cells expressed lower CPT1C levels when subjected to Aβ_25-35_ stimulation.

2. CPT1C participates in Aβ_25-35_-induced oxidative stress and apoptosis, along with reducing the expression levels of AD-related markers.

3. Aβ_25-35_ could inhibit PPARα to decrease CPT1C expression, thereby involving in AD pathology.
